# Ileocolic Intussusception - A rare cause of acute intestinal obstruction in adults; Case report and literature review

**DOI:** 10.1186/1749-7922-3-26

**Published:** 2008-08-04

**Authors:** Muhammad Najm Khan, Avi Agrawal, Paul Strauss

**Affiliations:** 1 Department of General Surgery, Royal Hospital Haslar, Gosport, UK; 2 Department of General Surgery, Darnet Valley Hospital, Dartford, Kent, UK

## Abstract

Colonic Intussusception although common in children, is a rare cause of acute intestinal obstruction in adults. The etiology, clinical presentation and management of this condition is different in adults as compared to children. Pre-operative diagnosis is usually difficult due to the non specific and intermittent nature of the symptoms. CT scan can be a helpful adjunct in establishing the diagnosis. We present a case report of adult ileocolic intussusception with classical radiological signs and operative findings. A brief literature review is also presented with emphasis on the controversy of reduction of the intussusception before resection.

## Case report

A 74 year old male presented with a four month history of colicky right upper quadrant pain, altered bowel habits and weight loss. He did not have any rectal bleeding. On examination there were signs of anaemia and a palpable mass in the right upper quadrant. Base line blood tests including FBC were normal. An abdominal USS showed a 5 × 7 cm ill defined bowel related mass in the right upper quadrant. The liver was normal.

**Figure 1:**
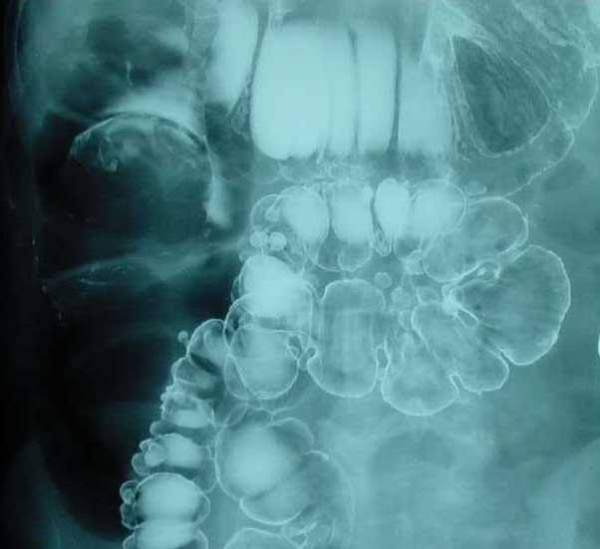
Barium enema showing a classical "claw sign".

**Figure 2:**
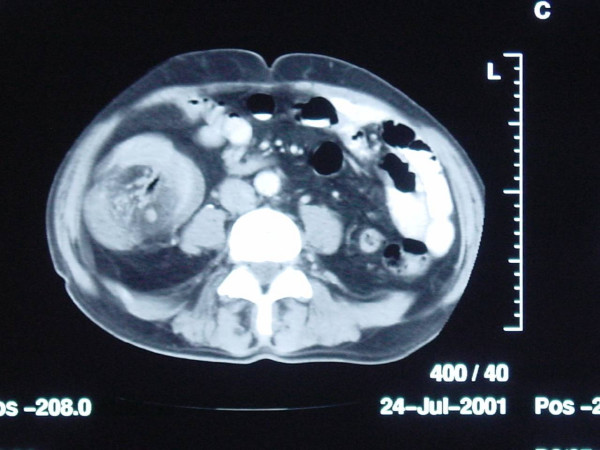
**CT scan of abdomen.** Red arrow demonstrates a "target lesion" diagnostic of intussusception.

A subsequent barium enema showed an intra-luminal filling defect measuring 8 × 10 cm in the proximal transverse colon with no flow of barium proximally (Figure 1). CT scan of the abdomen showed significantly thickened bowel loops with fat density within a proximal segment and a target lesion, consistent with a neoplasm or a lipoma causing Intussusception (Figure 2).

**Figure 3:**
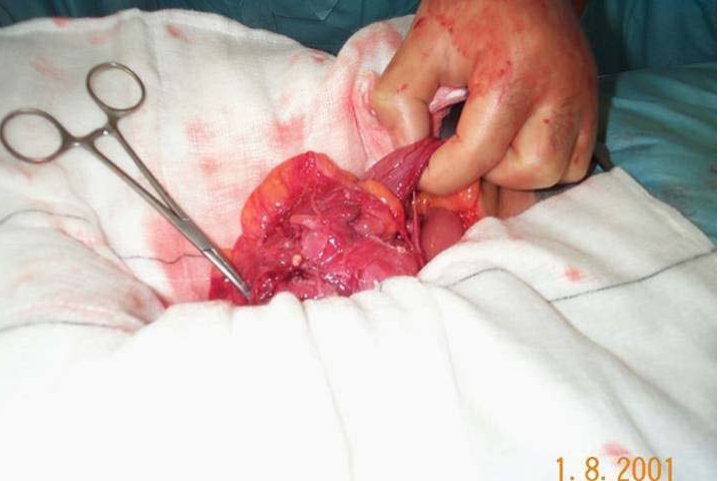
Operative picture demonstrating ileocolic intussusception.

**Figure 4:**
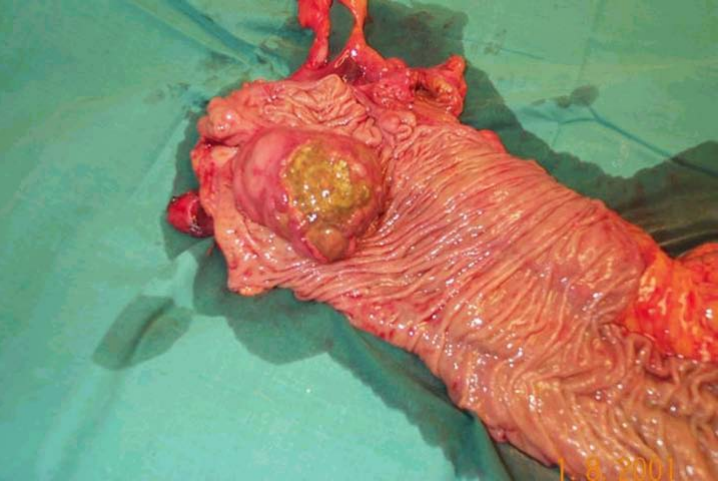
Resected specimen opened up to show the presence of intussusception.

A provisional diagnosis of colonic carcinoma was made although the tumour markers including CEA and CA 19.9 were normal. The patient underwent a laparotomy where an ileocolic intussusception was found at the level of hepatic flexure (Figure 3). A right hemicolectomy was carried out with a hand sewn end to end ileocolic anastomosis. The specimen was opened to reveal a protruding polypoidal mass in the caecum (Figure 4). The patient made an uneventful post operative recovery and was discharged seven days later. The histology of the specimen showed this to be a benign submucosal lipoma of the caecum protruding into the caecal lumen.

## Discussion

Intussusception is an uncommon cause of intestinal obstruction and more than 95% of cases occur in the paediatric age group [1]. Intussusception in adults is a rare pathology its incidence is around 2–3 per 1000,000 per year [2]. Due to this rare nature of the disease there are no large scale/multi-centre studies or meta-analyses published to investigate the management of adult intussusception. The aetiology, presentation and management of intussusception in adults is different from children. In children intussusception is usually idiopathic or secondary to a viral illness. However in adults in more than 90% of cases a lead point can be identified causing the intussusception [3,4]. This is usually a polyp or a tumour and in majority of these cases the colonic tumours are malignant [5,6].

The clinical presentation is very non-specific which makes this a difficult condition to diagnose. Abdominal pain, nausea, diarrhoea and bleeding per rectum are the common symptoms. Rarely this can present with acute intestinal obstruction. The classical triad of abdominal pain, sausage shaped palpable mass and passage of red current jelly stools seen in children is rarely observed in adults [6,7]. The use of investigations including a barium enema, ultrasound scan, and computed tomography can be helpful to establish the diagnosis [2,3,7-9]. CT scan has been reported to have a diagnostic accuracy of around 80% [10]. The classical finding on a CT scan is a target lesion or target sign which represents the outer intussuscepiens and the inner intussusceptum (Figure 2, 3). The dense intussuscepted mass comprising of swollen bowel and mesentery within the lumen of the bowel is responsible for the characteristic target lesion seen on the CT scan [7,11,12]. Ultrasound scan is a less invasive and reproducible investigation. The classical features include a donut sign in transverse view and pseudo-kidney sign on longitudinal view [13]. The examination is of limited value in the presence of significant amount of air in the intestine. A few studies have reported the use of colonoscopy in preoperative diagnosis particularly in the cases presenting with symptoms of large bowel obstruction [14]. However the examination is technically challenging and the diagnosis is difficult to make.

Benign lesions account for almost 25% cases of intussusception in adults. The commonest benign lesion is a lipoma in the colon. These are solitary submucosal lesions with 75% occurring in the right colon. Small lipomas are asymptomatic. Other benign lesions include adenomatous polyps and Peutz-Jeghers polyps. However in more than two thirds of cases there is a malignant tumour in the colon or small bowel resulting in intussusception [15-17].

Operative intervention is required in all cases of adult intussusception and unlike children conservative treatment does not work [2,6]. This usually involves segmental colonic resection. The optimal treatment for adult intussusception is slightly controversial. The type of procedure depends upon the location of intussusception, pre-operative diagnosis and condition of the intestine at the time of laparotomy. A few authors have described intra-operative reduction of intussusception before resection [5]. However most authors do not recommend this due to a higher incidence of malignancy in these cases [2,6-8,18,19] and hence the risk of tumour embolisation and seedling.

In most cases of adult colonic intussusception, primary resection without reduction should be performed particularly in those more than 60 years of age due to a higher risk of malignancy. In cases of small bowel intussusception reduction before resection should be carried out only if there is a pre-operative diagnosis of benign etiology, the bowel is viable or it entails resecting massive lengths of small bowel with the risk of short gut syndrome [12,20].

## Conclusion

Intussusception is a rare cause of acute abdomen in adults. A high index of suspicion and appropriate investigations (USS, Barium enema and CT scan) can result in prompt diagnosis. Unlike children 75% of cases are due to a malignant tumour in the small bowel or colon. The extent of resection and operative technique depend upon the age of the patient, results of investigations (benign or malignant) and the length of the bowel involved.

## Authors' contributions

MNK carried out the literature review and drafted the paper. AA designed the paper, literature review and reviewed the manuscript. PS literature review, provided the figures and reviewed the manuscript. All authors have read and approved the final manuscript.

## Consent

Written informed consent was obtained from the patient for publication of this  case report and accompanying images. A copy of the written consent is available  for review by the Editor-in-Chief of this journal.
